# Gut Microbiota Alterations in Patients with Panic Disorder: A Case-Control Study

**DOI:** 10.3390/nu17172772

**Published:** 2025-08-27

**Authors:** Tomasz Grąźlewski, Jolanta Kucharska-Mazur, Jerzy Samochowiec, Artur Reginia, Paweł Liśkiewicz, Anna Michalczyk, Błażej Misiak, Mariusz Kaczmarczyk, Ewa Stachowska

**Affiliations:** 1 Department of Psychiatry, Pomeranian Medical University, 71-460 Szczecin, Poland; 2 Department of Psychiatry, Wroclaw Medical University, 50-367 Wroclaw, Poland; 3 Department of Biochemical Sciences, Pomeranian Medical University, 71-460 Szczecin, Poland; 4 Department of Human Nutrition and Metabolomics, Pomeranian Medical University, 71-460 Szczecin, Poland

**Keywords:** gut–brain axis, panic disorder, anxiety disorders, gut microbiota, microbial diversity

## Abstract

**Background/Objectives:** Recent evidence suggests that gut microbiota plays an important role in anxiety and stress-related disorders through interactions along the gut–brain axis. Our aim was to determine the microbiological diversity of intestinal microorganisms in individuals with acute and remission phases of PD when compared to healthy individuals. Another aim was also to analyze the differences in the metabolic pathways occurring in the intestinal microbiota of individuals from the three analyzed groups. **Methods:** A diagnosis was established using the Mini-International Neuropsychiatric Interview (M.I.N.I). The gut’s microbiota composition was analyzed through bacterial 16S rRNA gene sequencing (V1–V2 regions). The clinical evaluations included a BMI measurement, Short Form-36 Health Survey (SF-36), Hamilton Anxiety Scale (HAM-A), Montgomery–Åsberg Depression Rating Scale (MADRS), Columbia-Suicide Severity Rating Scale (C-SSRS), and State-Trait Anxiety Inventory (STAI). **Results:** We recruited 62 participants (31 PD and 31 controls). After conducting quality control filtering, data from 54 participants were analyzed (25 PD, 11 acute, 14 remission, and 29 controls). Observed richness was lower in the acute PD (63) group than in the control (74) and remission (66) (*p* = 0.038) groups, whereas the Shannon and Simpson indices and beta diversity (PERMANOVA) were not significantly different. The Ruminococcus gnavus group was enriched in acute PD; no other deconfounded differences in microbial composition were detected. Predicted functional differences were detected by edgeR only and included the pathways that are related to steroid biosynthesis and innate immune signaling. **Conclusions:** Distinct gut microbial signatures were associated with PD, implicating both the metabolic and inflammatory pathways in disease pathophysiology.

## 1. Introduction

Panic disorder (PD) is a common mental disorder that is characterized by recurrent, unexpected episodes of severe anxiety, which are typically known as panic attacks [1]. A panic attack is defined as a sudden recurrent episode of intense fear that peaks within minutes, usually resolves within 10–20 min, and includes both physiological and psychological symptoms such as palpitations, chest pain, choking sensations, dizziness, and feelings of unreality (depersonalization or derealization) [1,2,3]. In addition to acute panic episodes, patients often develop anticipatory anxiety—a chronic worry about future attacks—and maladaptive behavioral changes such as an avoidance of situations that are associated with panic. PD has a lifetime prevalence of approximately 3%, predominantly affects young adults, and is about twice as common among women as men [4,5]. As a result, PD often causes marked impairment: patients may restrict their daily activities, experience difficulties at work and in relationships, and suffer a reduced quality of life. Individuals who are diagnosed with PD use medical services considerably more often than people without the disorder, resulting in a significant increase in healthcare costs and resource utilization [6,7,8].

In recent years, growing evidence highlights the link between gut health and mental health, particularly emphasizing the role of gut microbiota in anxiety and stress-related disorders. The gut microbiota, a vast community of microorganisms residing in the gastrointestinal tract, significantly influences the host’s physiology, including brain function and reactions to stress [9,10,11]. Communication between gut microbes and the central nervous system occurs along the microbiota–gut–brain axis, which integrates neural, endocrine, immune, and metabolic pathways [10]. Gut microbes regulate the production of neurotransmitters and their precursors (e.g., serotonin, GABA, tryptophan, etc.), and can secrete and upregulate essential metabolites, such as short-chain fatty acids (SCFAs) and brain-derived neurotrophic factor (BDNF), thus influencing cognitive functions and mood regulation [12,13]. Moreover, gut bacteria may influence the activity of the hypothalamic–pituitary–adrenal (HPA) axis, a main system that is responsible for stress responses. Dysregulated HPA axis signaling, which is commonly associated with high levels of cortisol and pro-inflammatory mediators, has been linked to anxiety disorders, potentially resulting from an altered gut microbial composition [14]. Importantly, panic disorder frequently co-occurs with gastrointestinal disorders, notably irritable bowel syndrome (IBS), with approximately 37% of PD patients fulfilling IBS diagnostic criteria, suggesting common underlying physiological mechanisms involving the gut–brain axis [2]. Although the exact mechanisms are not yet fully understood, a growing body of evidence points towards an interplay among endocrine, neurotransmitter, and microbiota disturbances [15], which eventually disrupts activity of the central nervous system (CNS), particularly in regions such as the amygdala, prefrontal cortex, and hippocampus [16,17], which play an important role in the pathophysiology of panic attacks. Findings from psychiatric conditions, such as anxiety and depression, along with evidence from animal studies, suggest that microbial pathways might play a pivotal role in the onset of psychiatric disorders [18,19,20,21]. However, the translation of promising preclinical findings into clinical practice for PD remains challenging due to the differences in microbiome–host interactions between animals and humans, and should be interpreted with caution. Studies specifically investigating microbiome composition in PD are limited. Patients with PD exhibit alterations such as a reduced abundance of beneficial butyrate-producing bacteria (Faecalibacterium, Coprococcus, and Roseburia) and increased relative abundance of potentially pro-inflammatory genera (Bacteroides) in the gut [22], along with increased diversity and elevated levels of Prevotella and Veillonella in the oral microbiome [23]. Emerging research also suggests that interventions targeting gut microbiota, such as probiotics or fecal microbiota transplantation, hold potential as novel therapeutic approaches for a variety of mental disorders, including anxiety disorders, by modulating the microbial signaling pathways involved in stress and immune regulation [24,25,26]. This raises the question of whether targeted modulation of the gut microbiota could indeed translate into clinically significant improvements in anxiety symptoms and stress responses among individuals with PD.

However, evidence that is specific to panic disorder remains limited, particularly studies comparing acute and remission phases and linking taxonomic shifts to predicted microbial functions. The present study addresses this gap by evaluating gut microbiota diversity and predicted functional pathways across acute PD, remission PD, and matched healthy controls.

The aim of this study was to compare gut microbiota diversity and composition in acute PD, remission PD, and matched healthy controls, and to assess predicted microbial functional pathways across these groups. We hypothesized that individuals with PD would show distinct intestinal microbiota profiles—differing in diversity, relative abundance, taxonomic composition, and predicted pathways—compared with the matched controls.

## 2. Materials and Methods

### 2.1. Patients

**Figure 1:**
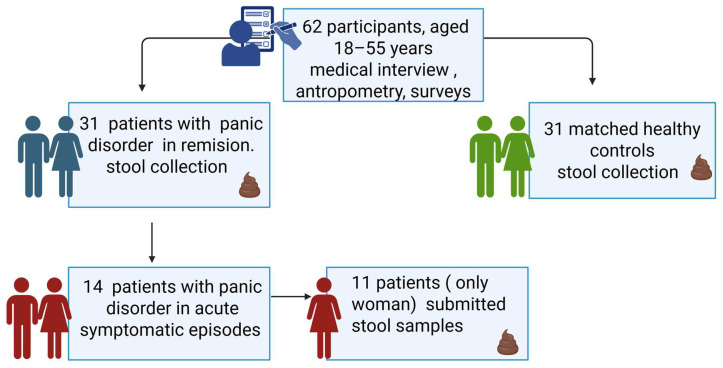
Research design graphic created in BioRender.

Between February 2019 and August 2020, patients with panic disorder were recruited from the outpatient psychiatry clinic of the Pomeranian Medical University in Szczecin (Figure 1). A control group consisting of age- and sex-matched healthy volunteers was also enrolled. All participants underwent a structured medical interview that was conducted by a psychiatrist. A diagnosis of PD was established using the full version of the Mini-International Neuropsychiatric Interview (M.I.N.I). Clinical characteristics were assessed using the Short Form-36 Health Survey (SF-36), Hamilton Anxiety Scale (HAM-A), Montgomery–Åsberg Depression Rating Scale (MADRS), Columbia-Suicide Severity Rating Scale (C-SSRS), and State-Trait Anxiety Inventory (STAI). Stool samples were collected by participants using a standardized medical device for fecal sampling. The Bristol Stool Form Scale (BSFS) was used to classify the stool types. Participants were recruited both during acute episodes and remission periods of PD. Acute states were identified by elevated anxiety scores (HAM-A > 25, STAI > 60), increased symptom intensity on clinical scales, and confirmed by psychiatric evaluation, whereas remission was characterized by reduced symptomatology and no PA in the last 4 weeks, which was verified through structured assessments. Patients were permitted to use selective serotonin reuptake inhibitors (SSRIs); however, the use of benzodiazepines and antibiotics within 4 weeks prior to stool sampling was an exclusion criterion. All participants completed a questionnaire regarding their current medical status and medical history, and provided written informed consent prior to inclusion in the study. Anthropometric parameters, such as height and weight, were measured using the bioimpedance method with an analyzer (Tanita SC-240MA, Tokyo, Japan), and the BMI was calculated according to the formula BMI = body weight (kg)/(height)^2^ (m). Information about the general dietary habits of the participants was also collected. No standardized dietary questionnaires were used in the study. Information on the patients’ dietary habits was obtained during a medical interview conducted during recruitment. All patients, both in the acute phase and in remission, described their diet as typical of the “Western” pattern. It was characterized by a regular, daily consumption of highly processed foods, red meat, and refined sugars, with occasional and low consumption of vegetables, fruits, whole grains, and good quality fats. Although these data have not been quantified using validated tools, their homogeneity among all participants provides important information about the nutritional background of the study groups. The study plan is presented in Figure 1.

The exclusion criteria included active substance use disorder within the past six months (except nicotine dependence), current or past severe organic brain injury, and cognitive impairment that is indicative of dementia. Additionally, patients were excluded if they presented with severe somatic illnesses, glucose intolerance, or current active inflammatory conditions. This study was approved by the Ethics Committee at the Pomeranian Medical University (Szczecin, Poland, approval number: KB-0012/11/19).

### 2.2. Sequencing Analysis of Bacterial 16S RNA Genes

DNA isolation from feces and sequencing of the V1–V2 regions of the 16S rDNA gene were performed using the Illumina MiSeq instrument (Illumina INC, San Diego, CA, USA) at the Institute of Clinical Molecular Biology, University of Kiel (Kiel, Germany), according to their own protocol (2 × 250 bp). DNA was isolated using microcentrifuge columns with a silica membrane. Extracted DNA was purified using an Agencourt AMPure^®^ XP instrument (Beckman Coulter, Brea, CA, USA).

Raw FASTQ files were processed using QIIME2 (version 2024.2). Samples with a low read count (<25,000) were discarded. After demultiplexing, the sequence counts per sample were as follows: minimum 29,304, median 53,179.5, mean 53,330.6, and maximum 83,367. Denoising of FASTQ sequences was carried out using the DADA2 plugin with the following parameters: (trunc_len_f = 200, trunc_len_r = 210, trim_left_f = 6, and trim_left_r = 6). In this step, 5711 amplicon sequence variants (ASVs) were retrieved, and the ASV frequency per sample was distributed as follows: minimum 19,121.0; 1st quartile 32,675.8; median 41,736.5; 3rd quartile 48,200.5; maximum 66,660.0; and mean 40,751.9. During the initial filtering step, features with a total frequency <10 (summed across all samples) were removed, resulting in 4542 ASVs for downstream analyses. Taxonomic assignment was performed using a naïve Bayes k-mer classifier that was pre-trained on the SILVA 138 (99% identity) full-length 16A rRNA gene reference database. Features not classified at the phylum level, or assigned to mitochondria, chloroplasts, archaea, or eukaryota, were removed. The alpha diversity indices were calculated on rarefied genus-level data (minimum depth of 17,880) using the *rtk* R library (version 0.2.6.1). Beta diversity was assessed using Bray–Curtis dissimilarity, computed with the *vegan* (version 2.6-10) R library on the same rarefied dataset.

### 2.3. Statistical Analysis

Alpha diversity metrics were compared between the groups using the Kruskal–Wallis rank-sum test. The Bray–Curtis distances were compared between the groups using Permutational Analysis of Variance (PERMANOVA) using the *vegan* package and were visualized via Principal Coordinate Analysis (PCoA). Differential abundance analysis (DAA) was carried out using a covariate-aware approach with the *metadeconfoundR* (version 1.0.2) R package. All statistical analyses were carried out using the R software (version 4.2.3, R Core Team (2022)).

## 3. Results

### 3.1. Baseline Characteristics

A total of 31 patients with PD and 31 healthy controls were recruited (Figure 1). Table 1 shows the basic parameters of patients obtained during patient recruitment for the study. Anthropometric measurements were performed by a clinical dietitian, and medical examinations, including the diagnosis of panic attacks, were performed by psychiatrists.

After conducting quality control filtering, we included 25 PD samples (11 in an acute state and 14 in remission) and 29 control samples in the final microbiome analysis. Six PD and two control samples were excluded due to low sequencing read counts (<25,000).

### 3.2. Gut Microbial Diversity

The results of the alpha diversity analysis of PD patients in the acute phase vs. remission vs. control showed the existence of taxon diversity, but only in one parameter (richness)—Table 2.

**Figure 2:**
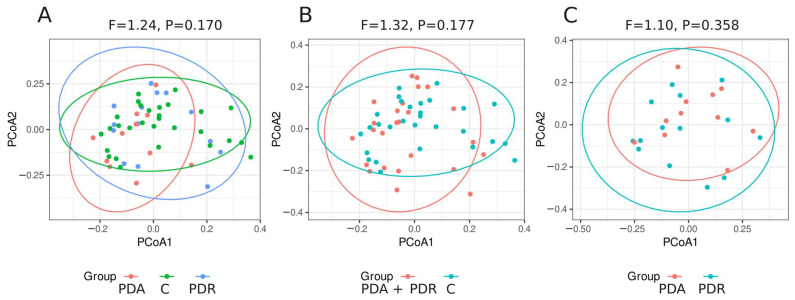
The results of the beta diversity analysis of patients with PD in the acute phase vs. in remission vs. controls. The Bray–Curtis beta diversity distances were subjected to Principal Coordinate Analysis and visualized in a two-dimensional space. Distances were compared using Permutational Analysis of Variance (PERMANOVA). Ellipses were constructed assuming a multivariate t-distribution at the level of 0.95. (**A**) patients with PD in the acute phase vs. in remission vs. controls. (**B**) patients with PD in the acute phase and in remission vs. controls. (**C**) control group. PDA—PD acute; PDR—PD remission; C—control.

The alpha diversity analyses (Shannon and Simpson indices) did not reveal any significant differences between patients in acute panic attacks, patients in remission, and the control group, with the sole exception of observed richness, which significantly differentiated patients in acute episodes from the other groups (*p* = 0.038). The beta diversity indices (Bray–Curtis) did not reveal any significant differences between the study group, regardless of the comparisons. (Figure 2).

### 3.3. Differential Abundance of Specific Taxa

**Figure 3:**
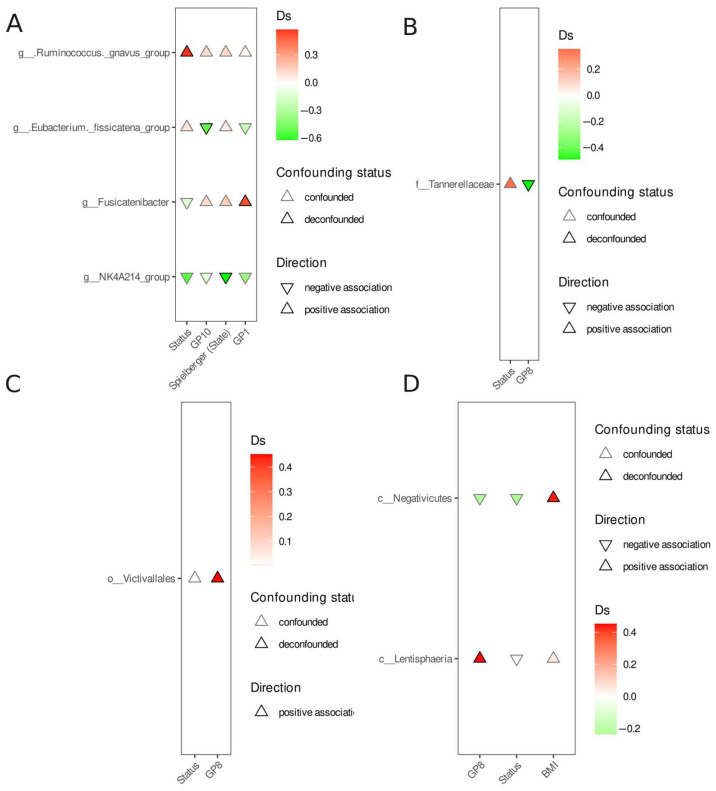
Differential abundance analysis—PD acute versus control. (**A**)—genus, (**B**)—family, (**C**)—order, and (**D**)—class. Each panel shows the microbial taxa that are associated with acute PD. Triangle direction: upward triangle (positive association) demonstrates taxa that are enriched in acute PD; downward triangle (negative association) demonstrates taxa that are enriched in the controls. The color intensity reflects the effect size (Cliff’s delta, Ds)—deeper colors correspond to stronger associations. Status—PD acute vs. control comparison. Confounding status: deconfounded (stroke outline)—highlights taxa for which the association signal is deconfounded, i.e., it cannot be explained by covariates (e.g., BMI, sex, age, SF-36, Spielberger trait, Spielberger state, and HAMA).

**Figure 4:**
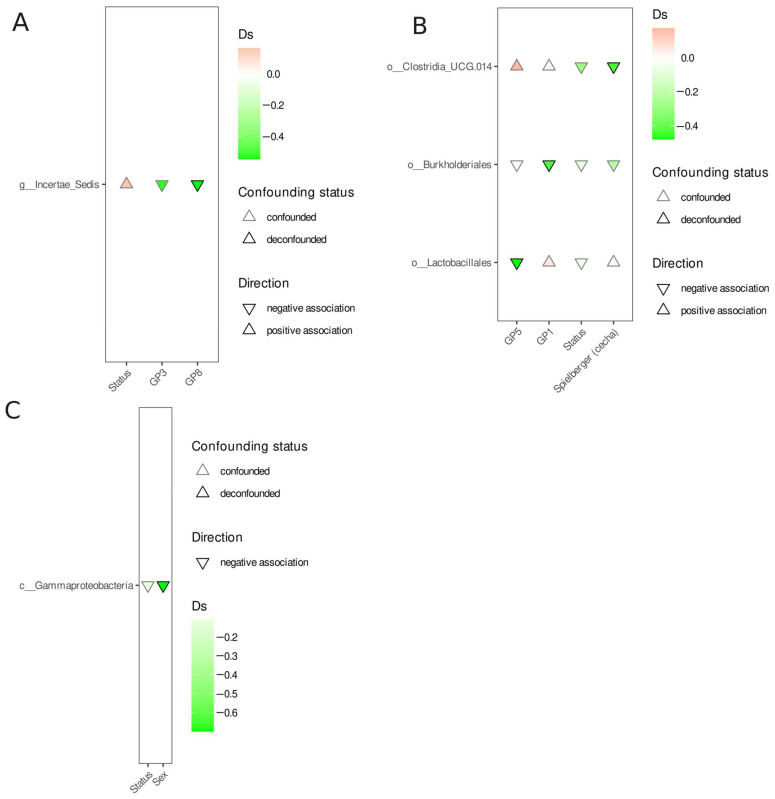
Differential abundance analysis—PD remission versus control. (**A**)—genus, (**B**)—order, and (**C**)—class. Each panel shows the microbial taxa that are associated with PD remission. Triangle direction: upward triangle (positive association) demonstrates taxa that are enriched in PD remission; downward triangle (negative association) demonstrates taxa that are enriched in the controls. The color intensity reflects the effect size (Cliff’s delta, Ds)—deeper colors correspond to stronger associations. Status—PD remission vs. control comparison. Confounding status: deconfounded (stroke outline)—highlights taxa for which the association signal is deconfounded, i.e., it cannot be explained by covariates (e.g., BMI, sex, age, SF-36, Spielberger trait, Spielberger state, and HAMA).

**Figure 5:**
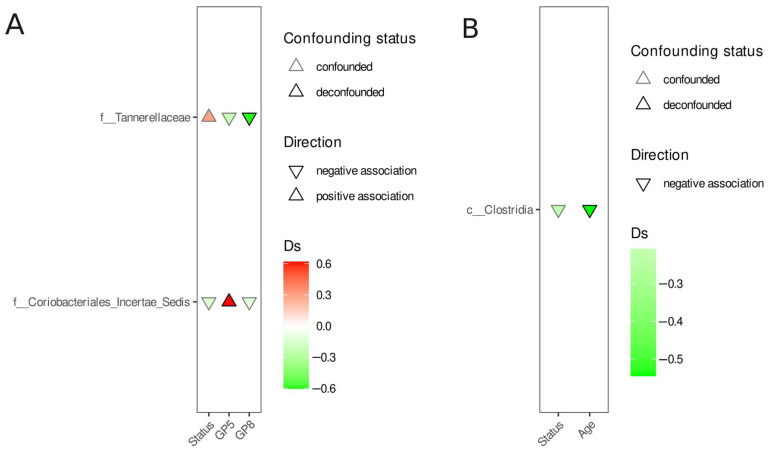
Differential abundance analysis—PD acute versus PD remission. (**A**)—family and (**B**)—class. Each panel shows the microbial taxa that are associated with PD acute or remission. Triangle direction: upward triangle (positive association) demonstrates taxa that are enriched in PD acute; downward triangle (negative association) demonstrates taxa that are enriched in PD remission. Color intensity reflects the effect size (Cliff’s delta, Ds)—deeper colors correspond to stronger associations. Status—PD acute vs. PD remission comparison. Confounding status: deconfounded (stroke outline)—highlights taxa for which the association signal is deconfounded, i.e., it cannot be explained by covariates (e.g., BMI, sex, age, SF-36, Spielberger trait, Spielberger state, and HAMA).

In order to identify differentially abundant features, we used a covariate-aware method in which any feature was considered differentially abundant if the association signal could not be attributed to any other covariate. The results of the comparisons between the PD acute and control, PD remission and control, and PD acute and PD remission groups are presented in Figure 3, Figure 4 and Figure 5. Except for the *Ruminococcus gnavus* group enrichment in the PD acute group when compared to the control (Figure 3), no other differentially abundant (and deconfounded) features were observed.

### 3.4. Predicted Microbial Functional Pathways

Predicted KEGG pathway analyses were run with six statistical methods; only edgeR identified between-group differences. For PD (PDA/PDR) versus the controls, edgeR identified the following: ko00513, ko00565, ko00624, ko00100, ko00945, ko05130, ko04622, ko00232, ko05100 (Table 3). For PDA versus PDR, edgeR identified: ko05142, ko00513, ko00624, ko00941, ko00100, ko00945, ko05219, ko00364, ko05130, ko05143, ko04614, ko05131, ko04622, ko00232, ko05100, and ko04075 (Table 4).

## 4. Discussion

### 4.1. Microbial Diversity

In our cohort, alpha diversity differed only for observed richness, with the most pronounced reduction in acute PD (compared with the control and remission groups), whereas the Shannon and Simpson indices showed no between-group differences. Microbial richness, defined as the total number of different bacterial species, is recognized as an important indicator of gut health. Our finding of reduced microbial richness in symptomatic PD patients aligns well with recent literature reviews and clinical observations that highlight decreased bacterial diversity in various anxiety disorders, including generalized anxiety disorder (GAD) [18,27,28] and PD [22]. Moreover, disturbances in microbiota composition are consistently reported as correlates of heightened anxiety symptoms [29]. Interestingly, in our study, microbiota richness was lower in panic disorder patients compared to the healthy controls, irrespective of their clinical state (symptomatic or remission). The lack of significant differences between symptomatic and remission phases suggests persistent microbiota alterations rather than a transient state-dependent phenomenon. However, we observed no differences in indices such as Shannon and Simpson between our cohort and the control group, and these results align with recent studies regarding GAD patients [18,29]. Additionally, we found no differences between groups in terms of beta diversity, which differs from prior research findings [18,22,29]. These discrepancies may be attributable to methodological differences, such as diverse analytical approaches or specific characteristics of our study population (e.g., medication status, dietary patterns, or severity of symptoms). Nevertheless, several intervention studies employing microbiota-targeted approaches, such as probiotics or dietary modifications, report a successful restoration of microbial diversity and composition, accompanied by notable improvements in psychiatric symptoms [14,30,31]. However, some studies report no clear differences in microbiota richness between patients with anxiety disorders and healthy controls, especially after controlling for confounding factors such as psychotropic medications or dietary habits [30,31,32]. Such a variability underscores the complexity of microbiome research and suggests that factors like medication, dietary patterns, chronic stress, and comorbidities significantly influence microbiota profiles, thus complicating the ability to obtain clear conclusions [33,34]. Further research with larger and methodologically homogeneous cohorts is needed to clarify the role and stability of microbiota alterations in panic disorder.

### 4.2. Ruminococcus gnavus Enrichment and Inflammatory Pathways

In our study, patients in the acute state exhibited increased abundance of *Ruminococcus gnavus*. *Ruminococcus gnavus* is an anaerobic, Gram-positive rod belonging to the Lachnospiraceae family. It colonizes the human intestine in early infancy, forming a permanent part of the microbiota. Its specialty is “nibbling” the terminal sugar residues of mucins thanks to its unique IT-sialidase.c [35] producing SCFA, propanol, and bioamines (tryptamine). In physiological amounts, *R. gnavus* helps maintain mucus renewal and provides colonocytes with short-chain fatty acids. However, when the microbiota loses its balance—for example, in Crohn’s disease, irritable bowel syndrome, or chronic stress—the species begins to dominate the intestine. An excess of its products (propionate, tryptamine, and glucorhamnan) promotes microinflammation and epithelial leakage, which can resonate in the immune and nervous systems. Hence, observations link the overgrowth of *R. gnavus* with the severity of anxiety, depression, and fatigue. In the intestines of patients with generalized anxiety disorder, there is a significant increase in *R. gnavus*. It has been noted that an increase in the number of these bacteria causes a decrease in the abundance of bacteria producing short-chain fatty acids (SCFA). These changes persisted despite a remission of the symptoms [18]. The mechanism by which *R. gnavus* influences mental disorders is unclear. A large multi-omics study, IBS-Dep, identified a strong association between an increase in the abundance of *R. gnavus* and a shift in tryptophan metabolism towards the neurotoxic kynurenine [36]. In our cohort, given that many PD patients were receiving SSRIs, which can affect serotonergic signaling and indoleamine metabolism, any microbiota-related inferences involving tryptophan/tryptamine/serotonin should be interpreted with caution, as medication effects cannot be excluded. Interesting observations related to the proliferation of *R. gnavus* were observed in patients with Crohn’s disease with coexisting depression/anxiety enrichment of *R. gnavus*; this was demonstrated by a correlation of its abundance with an unfavorable bile acid profile and higher Self-Rated Depression Scale (SDS) and Self-Rated Anxiety Scale (SAS/SDS) scores [37].

Collectively, these findings highlight the complex role that gut microbiota may play in psychiatric and somatic disorders, suggesting pathogenic mechanisms. Further research is needed to clarify these relationships, ideally employing larger, longitudinal cohorts, as well as a consistent methodology, integrated approaches combining microbiome analysis with clinical symptomatology, inflammation markers, and neurotransmitter profiling. Such studies could help clarify the clinical implications of microbiota shifts, paving the way toward targeted microbiome-based therapeutic strategies. The observed microbiota alterations reinforce patterns that are seen in anxiety and depressive disorders, characterized by an increased abundance of inflammatory taxa. These microbial shifts may contribute to chronic low-grade inflammation and neurotransmitter imbalance within the gut–brain axis. Such inflammatory states can activate stress-related pathways, including hyperactivation of the HPA axis, thereby exacerbating psychiatric symptoms characteristic of MDD (major depressive disorder) and GAD [33].

### 4.3. Microbiota Functional Pathways

Our metagenomic predictions indicated disruptions in both metabolic and immunological pathways of the microbiota–gut–brain axis; however, it is important to note that we used six different statistical approaches for pathway analysis; only one method (edgeR) detected any significant pathway differences between groups, while the others did not detect changes. Thus, the pathway results should be interpreted with caution. Our analysis suggested alterations in the steroid biosynthesis pathway (ko00100). Given the recognized role of gut microbiota functions as a “virtual endocrine organ” capable of modulating host steroid metabolism [38], it may affect circulating levels of neuroactive steroids (e.g., cortisol or sex hormones), thereby sensitizing the stress response systems. Indeed, recent findings show that dysbiosis can impair steroid hormone synthesis—children with ASD (autism spectrum disorder) exhibit reduced circulating estradiol and adrenal steroids that are linked to microbial changes [39]. Li Shao et al. [39] implicated a tumor necrosis factor (TNF-α)-driven increase in sphingolipid metabolism that inhibited steroid biosynthesis, highlighting a mechanism by which microbiota-induced inflammation might destabilize HPA axis feedback.

Parallel to these metabolic changes, we identified enrichment of the RIG-I-like receptor signaling pathway (ko04622), an innate immune pathway typically involved in antiviral responses. Chronic activation of these receptors, known to stimulate Type I interferon responses, may contribute significantly to neuroinflammation. For example, post-mortem brain analyses from individuals with major depression have revealed increased expression of the RIG-I signaling pathway genes in the hippocampus [40]. Such persistent immune activation in the gut or bloodstream may prime microglia and elevate interferon and cytokine levels, driving the neuroimmune dysregulation observed across anxiety and mood disorders.

Furthermore, our analyses indicated enrichment of pathways related to bacterial invasion of epithelial cells (ko05100) and pathogenic *Escherichia coli* infection (ko05130), suggesting increased abundance of Gram-negative pathobionts with invasive and pro-inflammatory potential, such as Enterobacteriaceae. Similar microbial patterns have been reported in schizophrenia, where elevated intestinal Enterobacteriaceae correlate with heightened systemic inflammation and compromised gut barrier integrity (“leaky gut”) [41]. Increased permeability of the gut barrier permits systemic translocation of bacterial endotoxins (lipopolysaccharide, LPS), activating toll-like receptors (TLRs) and inducing cytokine release. Such low-grade endotoxemia has been documented in both depression and ASD [42,43], and likely also plays a role in panic disorder pathophysiology.

This proposed model aligns closely with the elevated levels of pro-inflammatory cytokines (e.g., IL-6, IL-1β, and TNF-α) observed in PD patients [44]. These cytokines not only induce acute-phase responses but also modulate neurotransmitter signaling and HPA axis activity [45]. For instance, IL-6 can penetrate the blood–brain barrier, directly affecting corticotropin-releasing factor (CRH) neurons. This may potentially increase cortisol secretion and perpetuate anxiety in a self-sustaining inflammatory stress loop [46].

Altogether, our findings reinforce the emerging recognition that specific gut microbiota shifts, ranging from altered steroidogenic capacity to increased microbial virulence and immunological signaling, contribute to neuroimmune dysregulation and enhanced HPA axis reactivity. This integrative mechanistic framework is consistent with growing evidence from generalized anxiety disorder, depression, and other psychiatric conditions [22,31,47].

### 4.4. Clinical Implications

Our findings have potential clinical implications. Interventions aimed at restoring intestinal eubiosis and barrier integrity could mitigate aberrant immune signaling at its origin. For example, certain probiotics, such as Lactobacillus and Bifidobacterium strains, have demonstrated efficacy in strengthening tight junction function, thereby reducing stress-induced cortisol levels and cytokine release [48,49,50]. Fecal microbiota transplantation and anti-inflammatory microbial cocktails have shown anxiolytic and anti-inflammatory effects in studies focused on ASD and depression [51,52,53]. However, larger, rigorously controlled clinical trials are necessary to confirm these promising initial results and to address key issues such as long-term safety, patient acceptability, and consistency of therapeutic outcomes.

By targeting the microbial pathways involved in steroid metabolism, innate immune sensors (e.g., RIG-I/Toll-like receptors), and pathogen-associated molecular patterns (e.g., endotoxin), within the gut, it may be possible to correct downstream neuroendocrine imbalances that contribute to psychiatric symptoms. The critical role of the gut microbiota–brain axis further highlights the importance of dietary interventions, given the powerful influence of diet on microbiome composition [54]. Epidemiological evidence consistently supports the protective effects of the Mediterranean diet against depression and anxiety across diverse age groups, potentially through components such as polyphenol-rich fruits and vegetables, high dietary fiber intake, beneficial fats (omega-3 fatty acids), and low processed food consumption [55,56,57,58]. Clarifying which specific dietary components drive these effects will enhance the precision and effectiveness of nutritional recommendations in clinical practice.

### 4.5. Limitations and Future Considerations

Several limitations should be recognized when interpreting these findings. First, the cross-sectional design precludes establishing causality between microbiota alterations and PD symptomatology. Further, longitudinal studies are needed to clarify whether microbiome changes precede the disorder or result from it. Second, the relatively small sample size might limit the statistical power. Third, while 16S rRNA sequencing allowed for robust identification at the genus level, it lacks the capability to reliably differentiate between closely related bacterial strains or capture the full spectrum of microbial functional genes. Metagenomic approaches or metabolomics methodologies may yield enhanced insights into microbial functionality. Fourth, microbiota-derived metabolites, inflammation biomarkers, and neuroendocrine measures (e.g., cortisol levels) were not directly assessed. Consequently, any inferred links between the observed microbiota changes and host inflammatory or HPA axis activity are speculative and unconfirmed. Fifth, we did not control for the potential effects of SSRIs on the gut microbiome; most patients were on antidepressants, which could influence the microbiota composition. However, this factor was inherently tied to the patient group and was difficult to disentangle. Lastly, the participants came from a single province, which may limit the generalizability of these findings. Future larger, multi-center studies incorporating multi-omics analyses are necessary to fully highlight the microbiome-brain interactions in PD.

## 5. Conclusions

We demonstrated differences in the gut microbiota composition between patients with PD and healthy controls. The influence of gut microbiota on PD seems to involve the metabolic and inflammatory pathways. This relationship appears reciprocal, as PD-related stress and activation of the HPA axis may further modulate gut microbial diversity and function. Future research, particularly larger prospective cohort studies, is essential to explore the complex interactions between gut microbiota and PD more comprehensively.

## Figures and Tables

**Table 1:** The baseline results for patients and control subjects at the start of the study. The results of the alpha diversity analysis of PD patients in the acute phase vs. in remission vs. control.

Characteristics	Acute PD N = 14 Mean (SD)	Healthy Control N = 31 Mean (SD)	PD Remission N = 17 Mean (SD)	*p*-Value
Smoking	11/14 (79%)	30/31(97%)	15/17 (88%)	0.104
SF-36	25 (32)	4 (3)	16(14)	<0.001
Spielberger–State (STAI-S)	45 (12)	28 (6)	32 (7)	<0.001
Spielberger–Trait (STAI-T)	56 (10)	31 (7)	43 (6)	<0.001
HAM-A total	..	1 (2)	11 (7)	<0.001
MADRS	10 (46)	0.2 (1.1)	1.7 (2.3)	<0.001

**Table 2:** The results of the alpha diversity analysis of patients with PD in the acute phase vs. in remission vs. controls. PDA—panic disorder acute; PDR—panic disorder remission.

Characteristic	Acute PDA N = 11 Mean (SD)	Healthy Control N = 29 Mean (SD)	PDR Remission N = 14 Mean (SD)	*p*-Value
Richness	63 (12)	74 (11)	66 (13)	0.038
Eveness	0.62 (0.05)	0.63 (0.05)	0.64 (0.05)	0.358
Shannon index	2.55 (0.33)	2.73 (0.28)	2.86 (0.27)	0.323
Simpson index	0.85 (0.04)	0.87 (0.04)	0.86 (0.05)	0.551

**Table 3:** Comparison of KEGG pathways—PDA/PDR versus control.

Method	Number of Features	Common Features	Different Features
limma voom	0	0	0
LinDA	0	0	0
ALDEx2 Welch’s *t*-test	0	0	0
DESeq2	0	0	0
edgeR	9	0	9
Maaslin2	0	0	0

Nine KEGG pathways were identified by edgeR: ko00513—various types of N-glycan biosynthesis; ko00565—Ether lipid metabolism; ko00624—Polycyclic aromatic hydrocarbon degradation; ko00100—Steroid biosynthesis; ko00945—Stilbenoid, diarylheptanoid, and gingerol biosynthesis; ko05130—pathogenic Escherichia coli infection; ko04622—RIG-I-like receptor signaling pathway; ko00232—Caffeine metabolism; ko05100—bacterial invasion of epithelial cells.

**Table 4:** Comparison of KEGG pathways—PDA versus PDR.

Method	Number of Features	Common Features	Different Features
limma voom	0	0	0
LinDA	0	0	0
ALDEx2 Welch’s *t*-test	0	0	0
DESeq2	0	0	0
edgeR	16	0	16
Maaslin2	0	0	0

Sixteen KEGG pathways were identified by edgeR as follows: ko05142—Chagas disease; ko00513—various types of N-glycan biosynthesis; ko00624—Polycyclic aromatic hydrocarbon degradation; ko00941—Flavonoid biosynthesis; ko00100—Steroid biosynthesis; ko00945—Stilbenoid, diarylheptanoid, and gingerol biosynthesis; ko05219—bladder cancer; ko00364—Fluorobenzoate degradation; ko05130—pathogenic Escherichia coli infection; ko05143—African trypanosomiasis; ko04614—Renin-angiotensin system; ko05131—Shigellosis; ko04622—RIG-I-like receptor signaling pathway; ko00232—Caffeine metabolism; ko05100—bacterial invasion of epithelial cells; ko04075—plant hormone signal transduction.

## Data Availability

The data are not publicly available due to privacy, legal, or ethical reasons. The data presented in this study are available upon request from the corresponding author.
